# Protective effects of *Ceratonia siliqua* extract on protamine gene expression, testicular function, and testicular histology in doxorubicin-treated adult rats: An experimental study

**DOI:** 10.18502/ijrm.v13i8.7507

**Published:** 2020-08-19

**Authors:** Hengameh Mehdi Khani, Mehrdad Shariati, Mohsen Forouzanfar, Seyed Ebrahim Hosseini

**Affiliations:** ^1^Department of Biology, Shiraz Branch, Islamic Azad University, Shiraz, Iran.; ^2^Department of Biology, Kazerun Branch, Islamic Azad University, Kazerun, Iran.; ^3^Department of Biology, Marvdasht Branch, Islamic Azad University, Marvdasht, Iran.

**Keywords:** Ceratonia siliqua, Doxorubicin, Protamine, Testicular injuries, Rat.

## Abstract

**Background:**

Spermatogenesis is a complex process that takes place under the influence of many different genes.

**Objective:**

The aim of this study was to investigate the possible effects of *Ceratonia siliqua* hydroalcoholic extract (CSHAE) on protamine gene expression, testicular function, and testicular histology in doxorubicin-treated rats.

**Materials and Methods:**

56 adult male rats with a age range of 2.5 to 3 months (210 ± 10 gr) were divided into seven groups (n = 8/each). A) Control group was left untreated; B) Sham group received 0.3 ml distilled water intraperitoneally, C) Negative control group received 3 mg/kg doxorubicin, intraperitoneally once a week for 28 days; and D) Positive control group received 600 mg/kg of CSHAE orally for 48 days; E, F, G) the experimental groups 1, 2, and 3 received 150, 300, and 600 mg/kg of CSHAE respectively orally, for 48 days, as well as 3 mg/kg doxorubicin once a week for 28 days. Hematoxylin-eosin staining was used in the histological study of testes, and enzyme-linked immunosorbent assay method was used in measuring serum levels of testosterone. Protamine gene expression was determined by real-Time PCR method.

**Results:**

The mean body weight, testicular weight, testicular volume, testosterone level (p = 0.022), the count of Leydig, spermatogonia, spermatocyte, and spermatid cells, as well as protamine gene expression (p = 0.008) were significantly increased in the experimental group 2 compared to the negative control group. The regeneration of testicular tissue was observed in the experimental group 2.

**Conclusion:**

CSHAE has protective effect on doxorubicin-induced testicular injuries.

## 1. Introduction

Sperm production and testosterone secretion are the two basic functions of the testes (1). Spermatogenesis is a complex process in mammals involving the division and differentiation of spermatogonial stem cells into adult sperm (2). Spermatogenesis occurs in the testis under the control of the secreted testosterone hormone, and the testicular secretory activity is controlled by the hypothalamus-pituitary-testis axis (3). The male reproductive system is responsible for the production, maturation, and transmission of sperm, and any structural and molecular abnormalities in this system may result in infertility (4).

Genomic DNA is heavily compressed in adult sperm, and this DNA compression and packaging is accomplished by the nuclear matrix and protamine (5). Protamines are the main and the smallest protein in the sperm nucleus, and their structures are constant in different species (6). These proteins play key roles in sperm chromatin, and their deficiency has negative effects on male morphology and fertility (7). The most significant role that protamine plays is condensing chromatin in the sperm head (8), which is critical in the proper packaging of paternal DNA (9). Protamine genes are grouped or clustered together and are specifically involved in the process of spermatogenesis, that is, the production of mature spermatozoa (10).

Doxorubicin is an anthracycline drug that is mostly prescribed for solid tumors and has deleterious effects on fertility (11). Doxorubicin can attenuate testicular fat and testicular fatty acids by apoptosis in rat, thereby reducing spermatogenesis and primary spermatogony and spermatid counts (12). It damages spermatogonia in immature mice, and causes irreversible damage to spermatogenesis in adults (13).

The *Ceratonia siliqua* (*C. siliqua*) tree belongs to the Fabaceae family (14). It is native to the Mediterranean region, and is found in southern Syria, India and many parts of the Eastern Mediterranean area including Iran (15). *C. siliqua* seed is a great source of bioactive compounds such as fiber, polyphenols, cyclitols, and low levels of fresh fat (16), and its fruit contains flavonoids, like quercetin, luteolin, and kaempferol (17). Beside, gallic acid has been isolated from the leaves and pods of the *C. siliqua* (18), and lupeol, genistein, and liquiritigenin have also been found in the *C. siliqua* extract (19). Also, its seed stem cells contain fatty acids including myristic acid, palmitic acid, oleic acid, linoleic acid, and arachidonic acid (20). Its pulp contains sugars, fats, proteins, amino acids, gallic acids, and phenols (21). Clinical trials related to *C. siliqua *have used its combination in the treatment of elevated cholesterol level (22). A limited number of studies address its importance in the treatment of diseases such as cancer and diabetes (23, 24). The compounds present in *C. siliqua* pods have potential protective effects in patients with hepatotoxicity due to inflammation and oxidative stress against dextran sulfate sodium (25). In addition, its leaf and pod extracts are able to induce apoptosis in hepatocellular carcinoma cell line (T1) after 24-hr treatment with maximal dose through direct activation of caspase3 pathway (26). Moreover, *C. siliqua *extract is effective against DNA damage in L1210 murine leukemia cell nucleus by its anti-toxic capacity (27). Ata and colleagues found that *C. siliqua* extract can have beneficial effects on sperm concentration in rabbits (28). In a study by Mahdiani and coworkers, it was reported that the sperm concentration and motility were increased in the *C. siliqua* recipient groups and decreased oxidative stress and inflammatory factors (29).

Due to the antioxidant properties of *C. siliqua*, we try to investigate the possible protective effects of *C. siliqua* fruit and pod hydroalcoholic extract on protamine gene expression, testicular function, and testicular histology in doxorubicin-treated rats. If it was found to be effective and can be used as a dietary supplement to reduce side effects of chemotherapeutic drugs including doxorubicin on male sex cells and aid in their recovery process.

## 2. Materials and Methods 

### Preparation of hydroalcoholic extract 

*C. siliqua *fruits were grown in and imported from Turkey. The dried fruit of *C. siliqua *was milled into powder form, 100 gr of the powder was soaked in 500 ml 70% ethanol for 72 hr in a percolator. The mixture was filtered and the solution was placed in a rotary apparatus to evaporate the solvent. The honey-like material left was placed in a desiccator attached to a vacuum pump to dry completely. Then, the desired doses of the extract were prepared by dissolving the appropriate amount of dried extract (150, 300, and 600 mg) in 1 ml of normal saline to be administered according to the animal weight (30).

### Animal treatment 

This experimental study was performed in April, 2018 at the Namazi Hospital of Shiraz. In this experimental study, 56 adult male Wistar rats (2.5 to 3 months old) weighing 210 ± 10 gr were used. They were randomly housed in standard cages under identical conditions at 20-22°C in 12-hr light/dark cycle. They were provided with sufficient water and dried food, and all ethical considerations regarding laboratory animals were met.

Animals were divided into seven groups (n = 8/each) as follows: the control group was left untreated; the sham group received 0.3 ml distilled water, intraperitoneally; the negative control group received 3 mg/kg doxorubicin, intraperitoneally, once a week for 28 days; the positive control group received the maximum dose (600 mg/kg) of *C. siliqua* extract, orally, for 48 days by gavage; the experimental groups 1, 2, and 3 received 150, 300, and 600 mg/kg of extract for 48 days by gavage, respectively + 3 mg/kg doxorubicin once a week for 28 days (31-33).

At the end of the experiment, animals were anesthetized with ketamine and xylazine (Rotexmedica GmbH, Germany), blood samples were taken from the left ventricle of their hearts, and their testes were removed and weighed separately by digital scales.

### Histological studies 

Testes were fixed in 10% neutral formalin. Alcohol dehydration was performed at different concentrations (from low to high). The clearing step was performed by placing the tissues in two containers containing xylene. In the replacement phase, tissues were placed in three containers of molten paraffin (65°C) for 1 hr each. The fixed testes were sectioned at 5-4 μm thickness, and the tissue sections were stained using hematoxylin-eosin (H&E) dye. In order to count the cells from each group, transverse sections of the seminiferous tubules were examined by light microscopy. At least five seminiferous tubules were selected from each slide. The numbers of spermatogonia, spermatocytes, spermatids, Sertoli, and Leydig cells present in lumen were compared between the different groups. All histological studies were performed under supervision of a pathologist (34).

### Testicular volume measurement

Testicular volume was measured by the amount of water displaced when the testicles were immersed in a cylinder of warm water (37°C) filled to the brim. Testicles were placed in a 2-L graduated cylinder with a known volume of water. The water displaced by testicles was then read to the nearest 5 ml to determine testicular volume (35).

### Testosterone measurement 

Blood samples were kept in vitro for 20 min and centrifuged at 5000g for 15 min. Their sera were removed from the clot with a sampler. Testosterone level was measured by enzyme-linked immunosorbent assay (ELISA) method (ELISA kit DR6, Germany).

#### Protamine gene expression 

In general, the following four steps were carried out to evaluate protamine gene expression:

1. RNA extraction by Biobasic Manufacturing Kit (EZ-10 Spin Column Total RNA Mini-Preps Kit.

2. DNase I treatment by Takara Manufacturing Kit (Cat. # 2215A).

3. Complementary DNA (cDNA) synthesis by Takara Company Kit (# RR037Q (Cat. (PrimeScript; RT Reagent Kit)).

4. Real-time polymerase chain reaction (PCR) by Takara Machine Bucket (Cat. # RR081A) Syber green 1 (36).

To begin with, the isolated sperms with testicular epididymis were wrapped in aluminum foil, put in a nitrogen flask, and transferred to a nitrogen tank (37). The RNA extraction was performed according to the RNA extraction kit instructions using the extraction kit solutions, and the manufacturer's proposed protocol. Following the RNA extraction, DNase and Takara Co. kit were used to remove genomic DNA (38).

Using the extracted RNA, cDNA synthesis was performed according to the kit instructions (Cat # RR037Q) based on reverse transcriptase enzyme. The synthesized cDNA was stored at -20°C to be used in real time PCR (39).

To measure the possible changes in the protamine gene expression, relative quantitative real-time PCR was performed using a kit (Takara Cat. # RR081A). The PCR process for each cDNA sample was carried out using both forward and reverse primers, and Master Mix (SYBRⓇ Premix). The reaction was initiated with temperature and time settings according to the kit protocol. Since the quality and design of primers is the most important factor affecting PCR efficiency, all primers were designed by Allele IDv7.8 software (Table I), and Beta-2-Microglobulin (B2M) gene was used as the internal control.

The basic requirements for gene expression evaluation were: (1) determining the PCR efficiency and standard curve to obtain identical duplication of fragments per cycle for the gene in question compared to the internal control, (2) determination of template concentration (cDNA) for subsequent experiments, and (3) to obtain a threshold number that shows the most appropriate PCR efficiency (40).

Real-time PCR efficiency is calculated using linear gradient obtained from CTs (cycle of threshold) differences.

After completing the thermocycler activity, viewing the graphs showing increased number of desired fragments and the rate of fluorescence emission, we measured the rate of change in gene expression relative to B2M gene and the control (lacking differentiating environments) by calculating ΔΔCt.

The lessΔΔCt = The more gene expression

**Table 1:** The primers used in the evaluation of protamine gene expression


**Genes**	**Primer sequences**	**Sizes (bp)**
	Forward: 5'-ATGGCCAGATACCGATGCTGC-3'	
**PRM1**	Reverse: 5'-CTCCTCCGTCTGCGACATCTTC-3'	80
			Forward: 5'- CGTGCTTGCCATTCAGAAA -3'	
**B2M**	Reverse: 5'**-**ATATACATCGGTCTCGGTGG -3'	244
	PRM1: Protamine 1, B2M: Beta-2-Microglobulin		

### Ethical consideration

All interventions in rats were done according to the protocol provided by the ethics committee of the Islamic Azad University Shiraz Branch (IR.IAU.SHIRAZ.REC.1398.028).

### Statistical analysis 

Histological, hormonal, and gene expression data were analyzed using the SPSS software version 22 and One-way ANOVA and Tukey test. Real-time PCR data were analyzed using either the ΔΔCT-2 or Livak method. P ≤ 0.05 was considered as statistically significance.

## 3. Results

The mean body weight, testicular weight, and volume and number of spermatogonia, spermatocytes, spermatids, and Leydig cells, testosterone level, and protamine gene expression (p ≤ 0.001) and Sertoli cells (p ≤ 0.05) showed a significant decrease in the negative control (receiving doxorubicin alone) group compared to the control and sham groups (Tables II, III).

Also, the mean body weight, testicular weight volume, number of spermatogonia, Leydig cells, testosterone concentration(p ≤ 0.001) and spermatocytes (p ≤ 0.05) in the experimental group 1 decreased significantly compared to the control and sham groups (Tables II, III).

The mean number of spermatid cells (p ≤ 0.001) was significantly increased in the experimental group 1 compared to the negative control group, however, it was significantly lower than the control and sham groups (Table II). No significant differences were observed in the mean of the Sertoli cell count and protamine gene expression level in the experimental group 1 compared to the control, sham, and negative control groups (Table III).

Mean testicular weight, testicular volume, and the number of spermatogonia, spermatocyte in the experimental group 2 significantly increased compared to the negative control group, while showing a significant decrease compared to the control and sham groups (Tables II, III). The mean number of spermatid cells was significantly increased in the experimental group 2 compared to the negative control group, however, it was significantly lower than the control and sham groups (Table III). The mean body weight, Leydig cell count, testosterone level, and protamine gene expression (p = 0.008) were significantly increased in the experimental group 2 compared to the negative control group (Tables II, III). The mean number of Sertoli cells in the experimental group 2 remained unchanged compared to the negative control, sham, and control groups (Table III).

The mean body weight, testicular weight and volume, number of spermatogonia, spermatocytes, spermatids and Leydig cells, testosterone concentration (p ≤ 0.001), and spermatocytes (p ≤ 0.05) Also protamine gene expression (p = 0.008) in the experimental group 3 (receiving doxorubicin and 600 mg/kg extract) decreased significantly in comparison with the sham and control groups (Tables II and III). The mean number of Sertoli cells in experimental group 3 did not change significantly compared to the negative control, sham, and control groups (Table III).

Finally, the mean body weight, testicular weight and volume, number of spermatogonia, spermatocytes, spermatids and Leydig cells, and testosterone level in the positive control group (receiving 600 mg/kg extract) rose significantly compared to the negative control group (Tables II and III). The mean protamine gene expression (p ≤ 0.001) in the positive control group (receiving 600 mg/kg extract) rose significantly compared to the negative control group. The mean protamine gene expression (p ≤ 0.001) in the positive control group elevated significantly compared to the control and sham groups (Tables II, III). The mean number of Sertoli cells in the positive control group did not change significantly compared to the sham and control groups (Table III).

### Interpretation of protamine gene expression results

**Figure 1:**
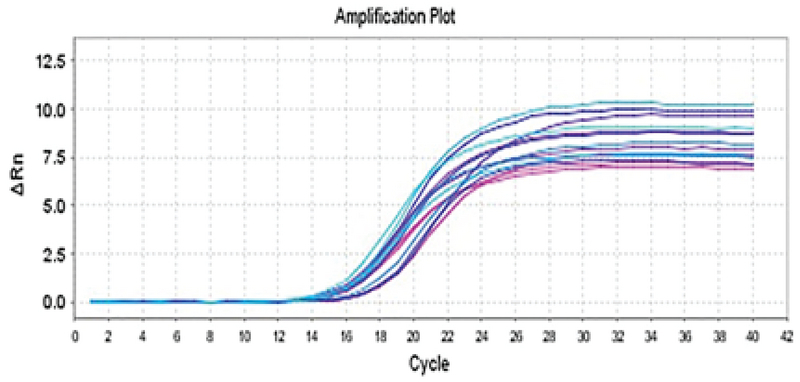
The protamine gene amplification curve. The number of cycles lies on the horizontal axis and the intensity of the fluorescent light emitted by the device lies on the vertical axis.

**Figure 2:**
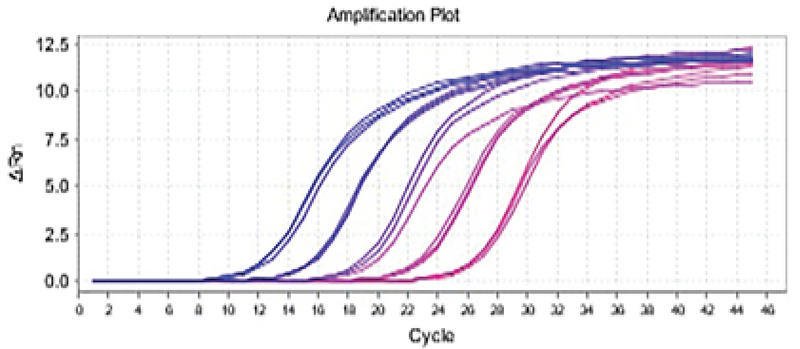
The B2M gene amplification curve. The number of cycles lies on the horizontal axis and the intensity of the fluorescent light emitted by the device lies on the vertical axis.

The protamine gene amplification curve was interpreted in different samples (experimental group 2 and treatment with doxorubicin and positive control) in real-time PCR reaction. The blue sample had a higher pattern of the protamine gene than the purple sample (negative control) at the start of the reaction. So, it enters the exponential phase sooner. The blue sample in cycle 12 (positive control), the bold blue sample (experimental group 2) in cycle 14, and the purple sample in cycle 16 enter the exponential phase. Since the blue (positive control) sample reached the exponential phase earlier and had a higher pattern, it could be said that the gene expression in the blue sample was higher (Figures 1, 2).

**Figure 3:**
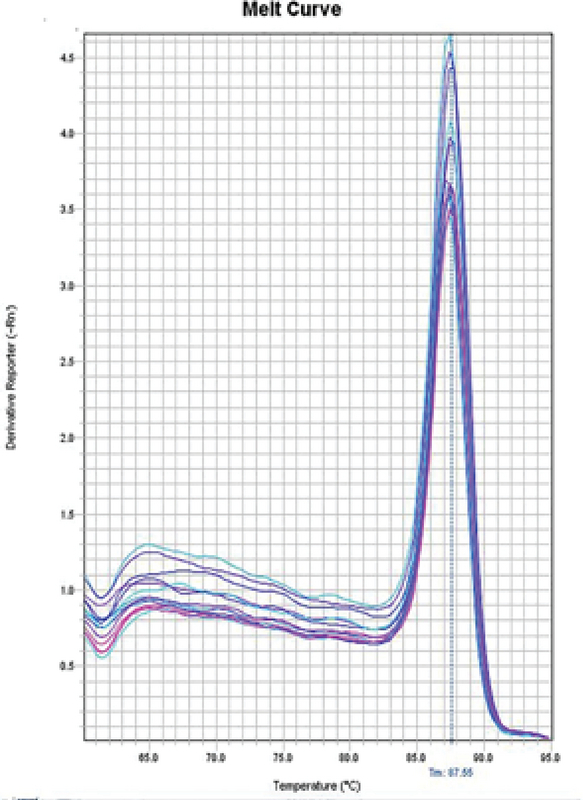
The protamine gene melting curve. This graph is plotted on the basis of the melting curve, and the position of the peak present shows 87.555°C; melting temperature (Tm) is the product of the B2M gene.

**Figure 4:**
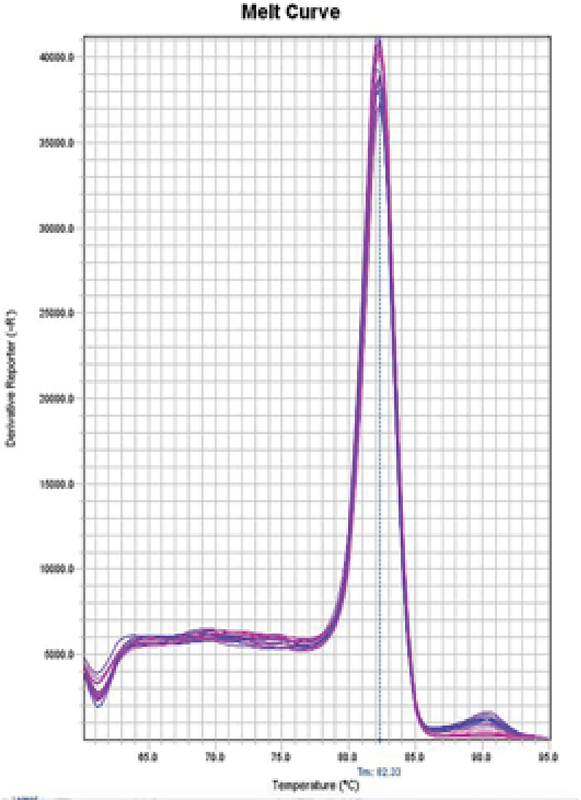
B2M gene fusion curve. This graph is plotted on the basis of the melting curve, and the position of the peak present shows 82.33°C; Tm is the product of the protamine-1 (PRM1) gene.

Examine the melt curve of the samples: If you use fluorescent dyes such as SYBR Green in real-time, you can test the melting curve for each sample. Since each gene has its own melting curve, therefore, the curves of a single gene in all samples must match. All curves must also be single-peak. Note that all peaks of a gene must be at the same temperature but the height of the peaks need not be the same (Figures 3, 4).

**Figure 5:**
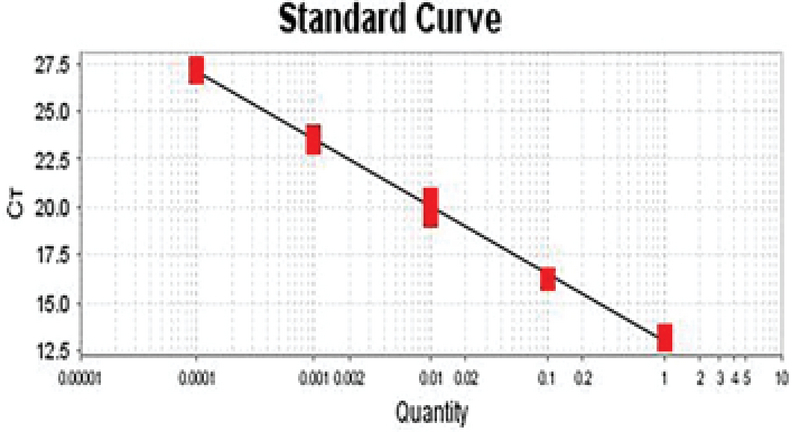
The standard curve for B2M gene. The slope of this curve is -3.534, and hence, the efficiency of the amplification reaction for gene is 92.203%.

**Figure 6:**
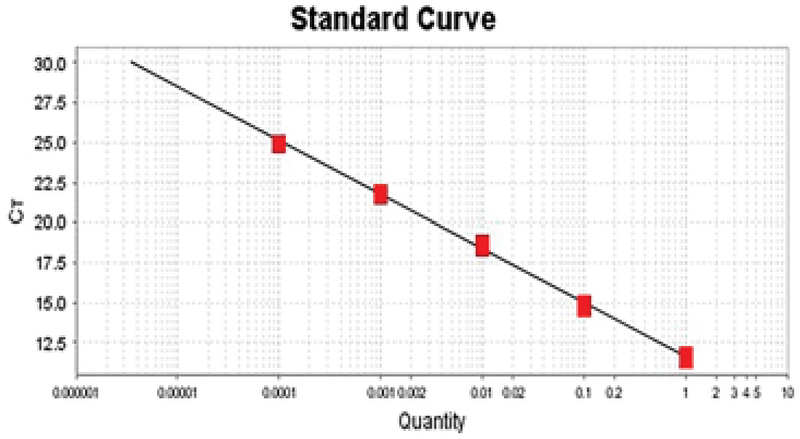
The standard curve for protamine gene. The slope of this curve is -3.384, and thus, the efficiency of the amplification reaction for PRM1 gene is 97.41%.

To draw a standard curve and calculate the amplification reaction efficiency after the end of the experiment, in most software the standard curve for each of the genes is plotted on the X-Y axis. In this logarithmic diagram, the sample pattern is on the X-axis and the cycle threshold (Ct) on the Y-axis. In standard curve diagrams, the slope of the line should be between 3.1-3.6 (Figures 5, 6).

### Histological results 

**Figure 7:**
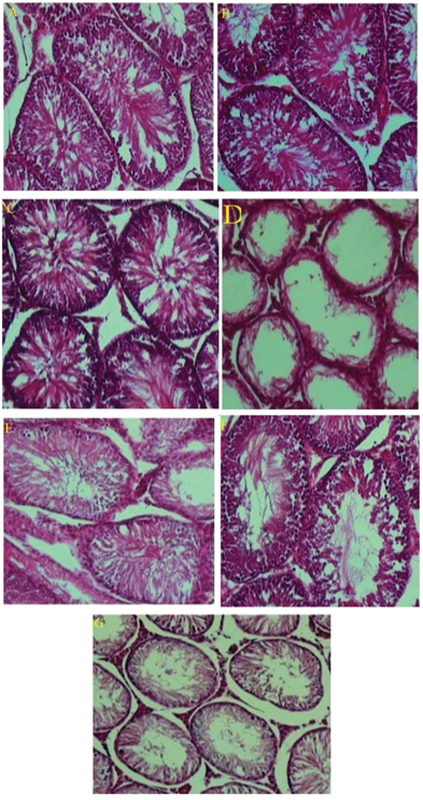
Photomicrographs of testicular tissue in different groups. (B) the control group; (D) the sham group; (F) the positive control group; (H) the negative control group; (J) the experimental group 1; (L) the experimental group 2; (N) the experimental group 3. Staining: H&E, magnification ×100.

There were no pathological changes in testicular photomicrographs of the control and sham groups, and testicular tubules and germ cells appeared normal (Figures 7A, B). Similar to the control and sham groups, tubules and germ cells were normal in testicular photomicrographs of the positive control group and tubules were filled with spermatogonia, spermatocytes, spermatids, and spermatozoa. Also, no pathological changes were observed in the testicular tissues of this group (Figure 7C). In contrast, wrinkled tubules, germinal epithelial fusion, and severe decrease in germ cell count were observed in testicular photomicrographs of the negative control group (receiving doxorubicin), and the presence of vacuoles inside the tubules indicated the deleterious effects of doxorubicin (Figure 7D).

In testicular photomicrographs of the experimental group 1, the deleterious effects of doxorubicin were still seen and the number of germ cells were decreased (Figure 7E). Conversely, greater reduction in wrinkled tubules were seen in testicular photomicrographs of the experimental group 2 compared to other experimental groups receiving higher and lower doses of the extract, indicating the positive effects of this dose on testicular regeneration (Figure 7F). The decline in the number of germ cells and the presence of vacuoles inside the tubules in testicular photomicrographs of experimental group 3 shows that high dose of *C. siliqua* hydroalcoholic extract (CSHAE) has no positive effect on testes (Figure 7G).

**Table 2:** Comparison of the mean body weight, testicular weight, testicular volume, and spermatogonial and spermatocyte cell counts in different groups


**Groups**	**Body weight (gr)**	**Testis weight (gr)**	**Testis volume (cm${}^{3}$)**	**Spermatogonia Cells (n)**	**Spermatocyte cells (n)**
**Control**	341.16 ± 27.67 a	1.73 ± 0.12 a	1.60 ± 0.11 a	82.20 ± 7.52 a	139.8 ± 4.11 a
**Sham**	338.33 ± 31.72 a	1.76 ± 0.22 a	1.64 ± 0.23 a	87.50 ± 9.02 a	131.16 ± 7.08 a
**Ceratonia 600 mg/kg**	332.42 ± 28.34 a (p ≤ 0.001)	1.75 ± 0.11 a (p ≤ 0.001)	1.65 ± 0.10 a (p ≤ 0.001)	91.22 ± 12.89 a (p ≤ 0.001)	133.44 ± 13.29 a (p ≤ 0.001)
**DOX**	252.57 ± 43.38 b (p ≤ 0.001)	0.72 ± 0.20 b (p ≤ 0.001)	0.59 ± 0.15 b (p ≤ 0.001)	33.00 ± 6.34 b (p ≤ 0.001)	51.75 ± 4.30 b (p ≤ 0.001)
**DOX + Ceratonia 150 mg/kg**	285.00 ± 22.41 bc (p ≤ 0.001)	0.83 ± 0.15 bc (p ≤ 0.001)	0.76 ± 0.14 bc (p ≤ 0.001)	35.33 ± 13.66 bc (p ≤ 0.001)	87.25 ± 6.22 bc (p ≤ 0.05)
**DOX + Ceratonia 300 mg/kg**	303.00 ± 15.91ac (p = 0.009)	0.94 ± 0.16 c (p ≤ 0.001)	0.85 ± 0.15 c (p ≤ 0.001)	49.88 ± 13.36 c (p = 0.046)	91.25 ± 10.61 c (p = 0.025)
**DOX + Ceratonia 600 mg/kg**	276.44 ± 21.36 bc (p ≤ 0.001)	0.68 ± 0.08 bc (p ≤ 0.001)	0.62 ± 0.08 bc (p ≤ 0.001)	29.75 ± 9.88 bc (p ≤ 0.001)	55.87 ± 3.88 bc (p ≤ 0.05)
The presence of at least one similar letter is indicative of no significant difference between the groups. Lack of similar letter between the groups indicates significant difference at the level of p ≤ 0.05. Values are shown as Mean ± SD. ANOVA test was followed by Tukey's test					

**Table 3:** Comparison of the mean number of Sertoli, Leydig and spermatid cells, testosterone concentration, and protamine gene expression in different groups


**Groups**	**Mean number of Sertoli cells**	**Mean number of Leydig cells**	**Mean number of spermatid cells**	**Testosterone (ng/ml)**	**Expression levels of Protamine mRNA**
**Control**	19.40 ± 2.13 a	19.00 ± 1.41 a	215.20 ± 14.29 a	1.37 ± 0.09 a	1.00 ± 0.05 a
**Sham**	19.16 ± 1.01 a	18.66 ± 2.15 a	208.83 ± 11.08 a	1.31 ± 0.18 a	0.89 ± 0.07 a
**Ceratonia 600 mg/kg**	19.22 ± 1.29 a (p ≤ 0.001)	19.77 ± 1.33 a (p ≤ 0.001)	232.11 ± 7.06 a (p ≤ 0.001)	1.34 ± 0.20 a (p ≤ 0.001)	4.07 ± 0.75 d (p ≤ 0.001)
**DOX**	12.00 ± 1.32 b (p ≤ 0.05)	6.12 ± 1.04 b (p ≤ 0.001)	81.37 ± 12.32 b (p ≤ 0.001)	0.096 ± 0.04 b (p ≤ 0.001)	0.28 ± 0.05 b (p ≤ 0.001)
**DOX + Ceratonia 150 mg/kg**	15.50 ± 1.54 ab	7.37 ± 1.08 bc (p ≤ 0.001)	133.87 ± 10.64 c (p ≤ 0.001)	0.41 ± 0.19 bc (p ≤ 0.001)	0.62 ± 0.15 abc
**DOX + Ceratonia 300 mg/kg**	17.37 ± 1.82 ab	14.37 ± 1.91 ac (p = 0.014)	151.25 ± 9.90 c (p = 0.006)	0.87 ± 0.09 ac (p = 0.022)	0.84 ± 0.11 a (p = 0.008)
**DOX + Ceratonia 600 mg/kg**	14.25 ± 1.25 ab	9.37 ± 2.41 bc (p ≤ 0.001)	54.50 ± 4.97 b (p ≤ 0.001)	0.33 ± 0.18 bc (p ≤ 0.001)	0.40 ± 0.07 bc (p = 0.008)
The presence of at least one similar letter is indicative of no significant difference between the groups. Lack of similar letter between the groups indicates significant difference at the level of p ≤ 0.05. Values are shown as Mean ± SD. ANOVA test was followed by Tukey's test					

## 4. Discussion 

According to the results of the present study, the mean body weight, testicular weight, volume, the number of spermatogonia, spermatocytes, spermatids, Sertoli and Leydig cells, serum testosterone level, and protamine gene expression declined significantly in the negative control group (receiving doxorubicin) compared to the control and sham groups, which is consistent with previous studies. It has been shown that doxorubicin induces spermatogenic damage in immature mice, and irreversibly impairs spermatogenesis in adults. These impairments are caused by injury to the blood-testicular barrier mediated by free radical generation and peroxidation (41). An administration of this drug to pre-adolescent rats also results in morphological and functional damage to Sertoli cells. It was demonstrated that after 21 and 28 days of treatment with 7.5 mg/kg doxorubicin, a significant decrease in testicular germ cells was observed, and semen producing segments, seminal tube height, and seminal tube diameter declined, and an increase in intra-tube segments was observed during the study period (42). These effects are not specific to cancer cells and can cause damage to healthy cells as well. Other studies have shown that the administration of doxorubicin to immature mouse leads to irreversible damage to Sertoli cells and induces severe disorder in spermatogenesis (43, 44). Such changes also occur in the testicular tissues of patients treated with doxorubicin (45, 46).

Based on our findings, the body weight, testicular weight, testicular volume, Leydig cell number, spermatogonia, spermatocytes, spermatid, testosterone level, and protamine gene expression were significantly higher in the experimental group 2 (receiving doxorubicin and 300 mg/kg extract) compared to the negative control group (receiving doxorubicin alone), but there was no significant difference in the number of Sertoli cells. The improvement observed in the testicular histology and the spermiogenesis of the experimental group 2 might be as a result of the antioxidant and antiapoptotic properties of *C. siliqua* plant (31, 47).

Although the mean body weight, all studied testicular parameters, and protamine gene expression in the positive control group (receiving 600 mg/kg extract) rose significantly compared to the negative control group, there was no such differences compared to the control and sham groups. However, protamine gene expression in the positive control group was significantly higher than the control group. It has been reported that the administration of hydroalcoholic *C. siliqua* seed extract increases sex hormone levels and sperm density in seminal tubes (28). Moreover, Soleimanzadeh and colleagues have found that *C. siliqua* fruit extract alleviated lead-induced reproductive toxicity in male mice. In this study, it was reported that a co-administration of CSHAE and Lead (Pb) significantly improved sperm parameters, glutathione content, antioxidant enzyme activity, and decreased the downregulation of testicular expression of nuclear factor erythroid 2-related factor 2 (Nrf2) and inducible nitric oxide synthase (iNOS) genes relative to the Pb control group (48).

Similarly, Vafaie and co-workers) showed that the consumption of 800 mg/kg *C. siliqua* extract for 35 days improves the sperm quality, biochemical parameters, thickening of testicular epithelium, and testosterone levels in busulfan-induced infertile mice (49). Also, malonaldehyde levels in the groups receiving extract were significantly lower than those of busulfan (a chemotherapeutic drug) group, while superoxide dismutase enzyme was significantly increased in these groups (49).

*C. siliqua* seed contains gamma-Linoleic acid and alpha-Linolenic acid, which can be converted to di-homogamma fatty acid and then to arachidonic acid (the precursor of all prostaglandins such as prostaglandin E2). It seems that arachidonic acid plays a major role in the testicular activities (50). Hossein and colleagues showed that arachidonic, oleic, and linoleic acids significantly enhance the motility of spermatozoa (51). These fatty acids also improve sperm acrosomal reaction at all incubation periods (51).

Furthermore, the increase in testosterone level can be justified by the direct effect of *C. siliqua *extract on Leydig cells, which play a key role in testicular hormone secretion (52). An important compound found in *C. siliqua* extract is genistein. Numerous studies have shown that genistein present in *C. siliqua* seed has protective effects on spermatogenesis (50-54). Verma and co-workers Found that genistein improves spermatogenesis by increasing lactate formation and antioxidant enzymes induced by insulin (53). That is why this compound can adjust the testicular dysfunction induced by type 2 diabetes in mice (53). In addition, Jalili and colleagues have shown that the administration of genistein can enhance spermatozoa quality and prevent morphine-induced side effects on sperm parameters (54). It has also been found that this compound relieves oxidative stress and spermatogenic injury induced by reperfusion and testicular ischemia by reducing the activity of testicular matrix metalloprotease (55). Chi and coworkers Showed that genistein effectively lowered intra-testicular testosterone level and stimulated spermatogenesis in rats treated with busulfan (56). Finally, it has been reported that genistein reduces gamma-ray-induced testicular injuries by antiapoptotic activity and remodeling spermatogenesis (57).

Another compound present in the extract of *C. siliqua* is gallic acid. Several studies have shown that gallic acid has protective effects on spermatogenesis (58, 59). Mehraban and colleagues showed that gallic acid can amend cyclophosphamide-induced reproductive toxicity in adult NMRI mice and increase the antioxidant capacity of testicular tissue (58). In another study, they also found that gallic acid has protective effects against sperm cell death as well as in vitro fertilization in adult male rats treated with cyclophosphamide (59).

*C. siliqua* extract also contains quercetin and luteolin, which have protective effects on spermatogenesis. For example, Ma and coworkers found that luteolin can amend testicular injuries and dysfunction through the Nrf2 signaling pathway by increasing regulation of Connexin 43 (Cx43) (60), and Khorsandi and colleagues reported that quercetin has some beneficial effects on spermatogenesis defects induced by titanium dioxide nanoparticles (61). Pretreatment with quercetin may increase testosterone concentration, decrease cell proliferation and oxidative stress in testicular tissue, and improve sperm quality as well as testicular tissue regeneration (61). Baltaci and coworkers (2016) showed that quercetin prevents arsenic-induced testicular injury through its antioxidant and antitumor effects (62). Similarly, Abd Ellah and colleagues found that this compound amends testicular injuries induced by di-(2-ethylhexyl) phthalate (63), and it can modify lambda cyhalothrin-induced reproductive toxicity in adult rats (64). Finally, Jahan and coworkers found that quercetin can possess potent therapeutic effects against bisphenol A-induced testicular toxicity in Sprague Dawley rats (65).

Based on the antiapoptotic and strong antioxidant (66) properties of *C. siliqua*, we conclude that this plant possesses the potential to protect subjects against doxorubicin toxicity through boosting activities of enzymes such as superoxide dismutase (SOX), Glutathione peroxidase (GPX), and catalase to protect against free radicals with cell toxicity (67). Since the moderate use of this plant has no apparent toxic effects, its beneficial potential in protecting against various toxicities is tempting.

## 5. Conclusion

Based on our findings, it appears that CSHAE at 300 mg/kg dose has a protective effect on doxorubicin-induced spermatogenesis toxicity, whereas its minimum (150 mg/kg) and maximum (600 mg/kg) doses have no significant influence on the number of germ cells, testicular tissue regeneration, and protamine gene expression. These results also indicate that the protective effects of *C. siliqua* extract are not dose-dependent, and even a dose as low as 300 mg/kg significantly increases the number of cells and regenerates testicular tissue injuries. Such protective effects are related to the antioxidant and antiapoptotic properties of various compounds present in the *C. siliqua *herb. Hence, this dose of CSHAE can be used as an effective substance against doxorubicin-induced testicular dysfunctions.

##  Conflict of Interest

The authors declare that they have no conflict of interests.
