# Corrigendum to “Protective effects of *Ceratonia siliqua* extract on protamine gene expression, testicular function, and testicular histology in doxorubicin-treated adult rats: An experimental study” [Int J Reprod BioMed 2020; 18: 667-682]

**DOI:** 10.18502/ijrm.v21i12.15044

**Published:** 2024-01-25

**Authors:** Hengameh Mehdikhani, Mehrdad Shariati, Mohsen Forouzanfar, Seyed Ebrahim Hosseini

The publisher has been informed of an error that occurred on page 667 in which the first authors name must be changed to Hengameh Mehdikhani. On behalf of the author, the publisher wishes to apologize for this error. The online version of article has been updated on 30 December 2023 and can be found at https://doi.org/10.18502/ijrm.v13i8.7507.

